# An efficient, localised approach for the simulation of elastic blood vessels using the lattice Boltzmann method

**DOI:** 10.1038/s41598-021-03584-2

**Published:** 2021-12-20

**Authors:** J. W. S. McCullough, P. V. Coveney

**Affiliations:** 1 Centre for Computational Science, Department of Chemistry, University College London, London, UK; 2 Informatics Institute, University of Amsterdam, Amsterdam, The Netherlands

**Keywords:** Biomedical engineering, Mechanical engineering

## Abstract

Many numerical studies of blood flow impose a rigid wall assumption due to the simplicity of its implementation compared to a full coupling with a solid mechanics model. In this paper, we present a localised method for incorporating the effects of elastic walls into blood flow simulations using the lattice Boltzmann method implemented by the open-source code HemeLB. We demonstrate that our approach is able to more accurately capture the flow behaviour expected in elastic walled vessels than ones with rigid walls. Furthermore, we show that this can be achieved with no loss of computational performance and remains strongly scalable on high performance computers. We finally illustrate that our approach captures the same trends in wall shear stress distribution as those observed in studies using a rigorous coupling between fluid dynamics and solid mechanics models to solve flow in personalised vascular geometries. These results demonstrate that our model can be used to efficiently and effectively represent flows in elastic blood vessels.

## Introduction

Many fields of computational biomedicine are moving towards the realisation of the virtual human. This concept embodies the creation of a high fidelity and personalised digital representation of the biophysical processes occurring within a particular individual. Such a model requires scalable computational methods that can study human-scale systems such as a particular organ but can also be efficiently coupled to simulations of other physiological components in order to build a virtual human. With continuing advances in computational performance, especially with the approach of exascale computers, the capacity to conduct high-fidelity human-scale simulations is becoming achievable. The development of a virtual human will allow clinicians to determine the optimal course of treatment for a given individual.

Numerical simulation of any physical system requires some level of approximation of the fundamental physics involved. A particular example of this is the assumption of rigid walls in the study of fluid flow through pipe-like structures. Whilst this is appropriate in many mechanical settings, organic structures such as blood vessels often exhibit elastic properties. Whether the use of a rigid wall approximation is valid for vascular simulations, or the implementation of an elastic wall model is necessary, depends on the particular vessels being studied, the scale of the simulation and the flow features of interest. Both options are used in practice and both come with benefits and drawbacks. In 1D models, where blood vessels are represented by a network of connected nodes, the effect of elastic walls can be implemented with a constitutive relation between pressure and the cross-sectional area of the vessel at solution locations^1–3^. The updated area can then inform the flow velocity at those sites. The implementation of elastic walls in a 3D model is significantly more difficult as it typically demands explicit coupling of the wall boundaries of the fluid domain to a solid mechanics model for an elastic vessel wall. Depending on the fluid solver used, the changing wall position may demand the fluid domain to be modified in response. Both of these procedures are usually regarded as being complex and computationally costly to implement in 3D and this is a reason why a number of 3D models utilise a rigid wall assumption^4–6^.

With a view towards creating virtual human-scale models, we are developing the capability to conduct full human simulations of arterial and venous vascular geometries using the open-source blood flow simulator HemeLB^6–12^. This solver has been specifically optimised to deal with the complex and sparse geometries characteristic of vascular domains. It exhibits excellent strong scaling characteristics on tens to hundreds of thousands of computer cores^6,13^ thanks to the fundamental properties of the lattice Boltzmann method (LBM) on which it is based. Such performance has been achieved through the spatial decomposition of complex vascular models at various sizes up to full human scale. These properties make HemeLB a good candidate for being able to simulate the blood flow in a virtual human model. All the simulations described in this work have been performed using the HemeLB code.

In this paper, we introduce a boundary condition for representing the effect of elastic walls at the edge of a lattice Boltzmann fluid domain. Our approach both retains the inherent scalability of the LBM and more accurately captures the key features of elastic flow without a loss of performance compared to a rigid wall implementation. The structure of our paper is as follows. In Numerical methods we outline the lattice Boltzmann method and the boundary conditions relevant to this work. We then compare the behaviour of our model against analytical results and those obtained with a rigid wall assumption. In Model application we use our model to simulate flow in personalised arteries of the left forearm. We then further discuss our simulation results and provide an outlook for how this work can be incorporated into a full-scale virtual human model. Our conclusions are summarised in the final section. In the Appendix, we provide computational details for the simulations conducted in this work.

## Numerical methods

In this paper we make use of the HemeLB code to solve 3D flow through a vascular geometry. In this section, we will give a brief outline of this approach followed by a description of our proposed method for replicating the impact of elastic walls. We then verify our implementation through comparison to analytical results that are relevant to blood flow simulation. In particular we will compare against results for Womersley flow in an elastic walled cylinder. This is suitable for comparison in that it resembles both the pulsatility that is characteristic of a heartbeat and explicitly allows for the impact of movable boundaries.

### The lattice Boltzmann method

Here we will give a brief introduction to the LBM; for a deeper discussion of the technique we refer the reader to the wider literature^14–18^. To describe a flow with the LBM, the domain is partitioned into a Cartesian grid with a constant spacing of $\Delta x$ in all 3D directions. At each nodal location, $\mathbf{x}$, a discrete set of values, $f_i(\mathbf{x} ,t)$, is assigned to represent the amount of fluid moving in direction *i* at time *t*. In this work, we use a D3Q19 model where fluid can stay at the current location or move to one of the 18 neighbours described by the sets: $i = 1{-}6$ $\left[ \left( \pm 1, 0, 0 \right) ,\left( 0, \pm 1, 0 \right) ,\left( 0, 0, \pm 1 \right) \right]$ and $i = 7{-}18$ $\left[ \left( \pm 1, \pm 1, 0 \right) ,\left( \pm 1, 0, \pm 1 \right) ,\left( 0, \pm 1, \pm 1 \right) \right]$. The flow described by $f_i(\mathbf{x} ,t)$ evolves over the time step $\Delta t$ with a single relaxation time operator:

1 $$\begin{aligned} f_i(\mathbf{x} +\mathbf{c} _i \Delta t,t + \Delta t) = f_i(\mathbf{x} ,t) -\frac{\Delta t}{\tau }(f_i(\mathbf{x} ,t)-f_i^{eq}(\mathbf{x} , t)). \end{aligned}$$

Here, $\mathbf{c} _i$ indicates the velocity set necessary to move flow to neighbour *i* in a single time step. $\tau$ relaxes $f_i(\mathbf{x} ,t)$ towards the equilibrium state $f_i^{eq}(\mathbf{x} , t)$, a discrete approximation of the Maxwell–Boltzmann distribution. As is demonstrated elsewhere in the literature, a Chapmann–Enskog expansion can be used to show that this framework represents the Navier–Stokes equation for fluid flow in a low Mach number limit. This expansion yields the expansion coefficients, $w_i$, for the equilibrium function,

2 $$\begin{aligned} f_{i}^{eq}(\mathbf{x }, t) = w_{i} \rho (\mathbf{x },t) \left( 1 + \frac{\mathbf{c }_{i}\cdot \mathbf{u }}{C_{s}^{2}} + \frac{(\mathbf{c }_{i}\cdot \mathbf{u })^{2}}{C_{s}^{4}} - \frac{|\mathbf{u }|^{2} }{C_{s}^{2}}\right) . \end{aligned}$$

For D3Q19 these are 1/3 for $i = 0$ (the source node), 1/18 for $i = 1{-}6$ and 1/36 for $i = 7{-}18$. $C_s$ represents the speed of sound of the fluid and evaluates to $\frac{1}{\sqrt{3}}$. Local macroscopic properties of density and momentum can be determined from moments of the $f_i(\mathbf{x} ,t)$ population as,

3 $$\begin{aligned} \rho (\mathbf{x} ,t) = \sum _{i}f_i(\mathbf{x} , t), \end{aligned}$$

and,

4 $$\begin{aligned} \rho (\mathbf{x} ,t)\mathbf{u} = \sum _{i}f_i(\mathbf{x} ,t)\mathbf{c} _i, \end{aligned}$$

respectively. Other relevant physical properties of pressure,

5 $$\begin{aligned} p(\mathbf{x} ,t) = C_s^2 \rho (\mathbf{x} , t), \end{aligned}$$

and viscosity,

6 $$\begin{aligned} \nu = C_s^2 \left( \tau -\frac{1}{2}\right) , \end{aligned}$$

arise from the Chapmann–Enskog expansion procedure and associated assumptions.

### Elastic wall theory

The analytical derivations of Womersley^19,20^ represents some of the seminal work in fluid flow through pipes. These two papers focus on idealised results for pulsatile flow through rigid and flexible pipes and provide a reference case that has been widely used as a verification model for computational fluid mechanics^11,21,22^. For a full derivation we refer to the source works or that of Figueroa^21^ or Filonova et al.^22^ but here we highlight equations of particular relevance to the later development of our elastic wall model. These field equations describe properties at a given radial, *r*, and axial, *z*, position in time, *t*, as a combination of steady and oscillatory components of flow. In the derivation of these equations, complex variables are used to simplify the expressions associated with the presence of oscillating flow, thus $i=\sqrt{-1}$. The oscillatory component is represented by the term associated with the $e^{i \omega \left( t - \frac{z}{c} \right) }$. In the cases where complex components appear in the final field equations, the output should be taken as the real part of the expression. The pressure field is described by:

7 $$\begin{aligned} p(r,z,t) = H e^{i \omega \left( t - \frac{z}{c} \right) } + p_0 + k_s (z-z_0), \end{aligned}$$

the axial component of velocity is given by:

8 $$\begin{aligned} w(r,z,t) = \frac{k_s}{4 \mu } (r^2 - R^2) + \frac{H}{\rho c} \left[ 1 - M \frac{J_0 \left( \frac{\Lambda r}{R} \right) }{J_0 \left( \Lambda \right) } \right] e^{i \omega \left( t - \frac{z}{c} \right) } , \end{aligned}$$

and the radial component of velocity is given by:

9 $$\begin{aligned} u(r,z,t) = \frac{H i \omega R}{2 \rho c^2} \left[ \frac{r}{R} - M \frac{2 J_1 \left( \frac{\Lambda r}{R} \right) }{ \Lambda J_0 \left( \Lambda \right) } \right] e^{i \omega \left( t - \frac{z}{c} \right) }. \end{aligned}$$

In these expressions, $J_n(x)$ is the *n*th order Bessel function of the first kind. Within these equations are parameters governed by the vessel and fluid being studied. Here *R* is the radius of the vessel, $z_0$ is the location of the entry of the cylinder whilst *c* is the wave speed within the elastic cylinder; $p_0$ represents the static background pressure whilst $k_s$ is the static pressure gradient. *H* represents the amplitude, and $\omega$ the frequency, of the oscillatory pressure. The Womersley number ($\alpha = R \sqrt{\frac{\omega \rho }{\mu }}$) is represented by the complex term $\Lambda = i^{3/2} \alpha$. The terms $\rho$ and $\mu$ represent the fluid density and fluid viscosity respectively. *M* is an elasticity factor derived from vessel and fluid properties.

### Proposed elastic wall boundary condition

Many existing representations of elastic walls within an uncoupled LBM simulation require the explicit changing of node types between fluid and solid to represent the change in wall location^23–25^. Whilst it is possible to take advantage of the inherent locality of LBM, this approach typically requires each lattice site to have a notion of how far away it is from the centre of the vessel. This is easy to achieve in simple representations of blood vessels as cylinders where the coordinates of the node can be used to deduce the local radial position. In patient-specific representations of blood vessels, this can become a much more challenging task as the vessel geometry and orientation can often deviate substantially from such simplifying assumptions. Similarly, the geometry and orientation of patient-specific vessels, combined with the often large number of lattice sites representing them, means that pre-computing such radial data is itself non-trivial. It is therefore advantageous to have a representation of elastic walls that does not fundamentally rely on knowledge of a site’s position within the vessel.

From a conceptual point of view, our approach assumes that the set of LBM fluid nodes represents the minimum fluid volume of the elastic vessel. We then implement a wall boundary condition that provides a non-zero fluid velocity at that location to mimic the effect of the vessel expanding beyond that point. For the generally small changes in vessel diameter^26^, combined with geometrical uncertainty in image-derived vascular simulation models, such an approach provides a useful compromise for capturing the effects of elastic walled vessels between simple rigid wall modelling and the complex and computationally expensive coupling to a solid mechanics model.

In our implementation, we build upon the Guo et al.^27^ (GZS) wall boundary condition. This method was selected due to its basis as an extrapolation condition—a similar concept to what we are trying to achieve. The GZS scheme was proposed as a method for representing curved boundaries. The non-equilibrium component of the distribution at the wall node is constructed from the neighbouring fluid node whilst the equilibrium component is constructed based on the desired location and characteristics of the curved boundary. For cases where the curved boundary is close to the wall (where $\Delta$, being the fraction of the unit cell the boundary is from the fluid node, is $\ge 0.75$), the wall node velocity is proposed by GZS to be $U_w = (U_{boundary} + (\Delta - 1) U_f)/\Delta$. This is used to construct $f_i^{eq}(\mathbf{x} _w, t)$ whilst $f_i^{neq}(\mathbf{x} _w, t)$ is taken to be the same as $f_i^{neq}(\mathbf{x} _f, t)$ in the post-collision construction of $f_i(\mathbf{x} _w, t) = f_i^{eq}(\mathbf{x} _w, t) + (1 - \tau ^{-1}) f_i^{neq}(\mathbf{x} _w, t)$.

**Figure 1:**
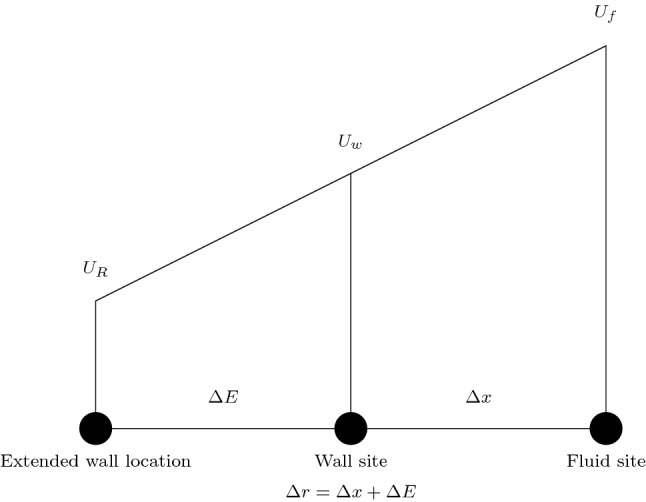
Schematic layout of fluid node, wall node and hypothetical wall location for the proposed boundary scheme. The extended wall location represents the position of the edge of the wall when it extends beyond the physically simulated domain.

In our boundary condition implementation, we consider the case of $\Delta = 1$ to apply a non-zero velocity at the wall node that is approximated to replicate the effect of an elastic boundary stretching beyond this point. The conceptual layout of this is given in Fig. 1.

If it is assumed that there is a linear change between $U_R$ and $U_f$, then $U_w = U_R + \frac{\Delta r - \Delta x}{\Delta r}( U_f - U_R)$. The $\Delta r$ term can be computed using common relations between pressure and the stiffness of the elastic wall. This expression for $U_w$ can then be fully derived in terms of the known value $U_f$ but taking into account analytical expressions for elastic vessels. Here we particularly look for the ratio between the edge of the elastic walled pipe and the location of the fluid node ${\Delta r}$ inside of it. For a ratio of $F = \frac{U_R}{U_f}$, the value of $U_w$ can be solely computed from $U_f$ as $U_w = \left( (\frac{F {\Delta x} + \Delta r - \Delta x}{\Delta r}\right) U_f$. For this computation we will only consider the oscillating part of the axial component of flow. At the extended wall location, the axial velocity is the real part of:

10 $$\begin{aligned} w(R,z,t) = \frac{H}{\rho c} \left[ 1 - M \right] e^{i \omega \left( t - \frac{z}{c} \right) } , \end{aligned}$$

whilst at the location of the fluid node it is the real part of:

11 $$\begin{aligned} w(R - \Delta r,z,t) = \frac{H}{\rho c} \left[ 1 - M \frac{J_0 \left( \frac{\Lambda (R- \Delta r)}{R} \right) }{J_0 \left( \Lambda \right) } \right] e^{i \omega \left( t - \frac{z}{c} \right) }. \end{aligned}$$

Combining these two expressions we get the following for the boundary velocity ratio *F*:

12 $$\begin{aligned} F = \frac{w(R,z,t)}{w(R - \Delta r,z,t)} = \frac{ 1 - M }{ 1 - M \frac{J_0 \left( \frac{\Lambda (R-\Delta r)}{R} \right) }{J_0 \left( \Lambda \right) } } = \frac{ 1 - M }{ 1 - M \frac{J_0 \left( \Lambda (1 - \frac{\Delta r}{R} ) \right) }{J_0 \left( \Lambda \right) } }. \end{aligned}$$

The value of F is dependent on the Womersley number ($\alpha$) of the local flow through $\Lambda$ and the extension of the flow $\Delta r/R$. $\alpha$ varies widely throughout the human vascular system from $O(10^{-3})$ in the capillaries to *O*(10) in the aorta. In our simulation efforts, the resolution of available human-scale domains means that we typically consider relatively large vessels where $\alpha >1$. Equally, within blood vessels, the amount of flow induced radial dilation is typically a relatively small value and often less than 10% of the radius^26^. Whilst *M* also varies based on vessel radius and Womersley number, its variation is much less than that of $\Lambda$ and $\Delta E$.

**Figure 2:**
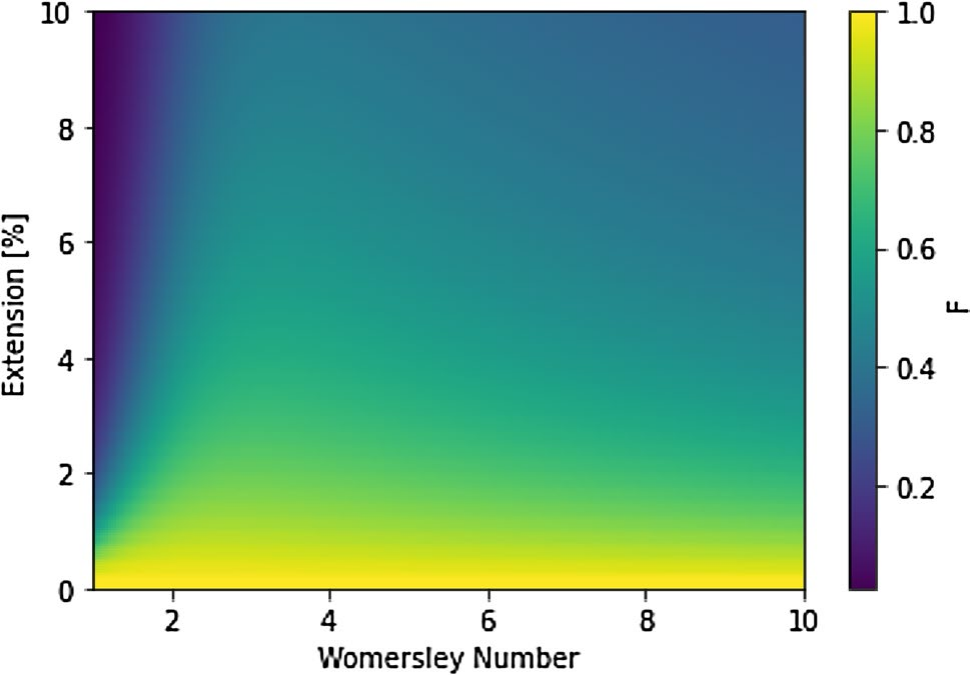
Distribution of boundary velocity ratio for a range of physiologically relevant values of $\Delta E$ (0–10%) and Womersley number (0–10) in vessels with a radius of 1 mm.

Based on these variations of parameters, we can generate a map of values of *F* for human vessels to apply for a given simulation. In cases where the variation in vessel characteristics is relatively small, this can help to narrow the selected value of F from this map. We precompute *F* for our boundary condition as the local Womersley number may not be known for a given boundary location within a large vascular tree. In Fig. 2 we illustrate the distribution of values of *F* for vessels of radius 1 mm.

## Model verification

The capability of the HemeLB code to capture Womersley flow in a rigid vessel has been demonstrated to very good accuracy for a range of Womersley numbers in the work of Nash et al.^11^. The purpose of the following section is to build upon this to examine how the new and existing boundary conditions are able to replicate the elastic wall conditions. In^21,22^, the problem of flow through the carotid artery is examined as a test case for an elastic wall model. We demonstrate how our model is able to capture the essence of elastic wall flow in a similar domain whilst not losing computational performance compared to a rigid wall model. The performance of our model is verified through the comparison to the analytical solutions for Womersley flow in an elastic vessel with a particular focus on the oscillatory component of flow. We consider a cylinder of *R* = 3 mm and total length of 4 cm. The vessel walls are set at a thickness of *h* = 0.1*R* and have material properties of: Young’s modulus *Y* = 20 kPa, Poisson ratio $\sigma$ = 0.5 and density of 1000 kg/m$^3$. We assume that the fluid has a density of 1000 kg/m$^3$ and viscosity of 0.004 Pa s. The applied oscillating pressure gradient has a period of $\frac{\pi }{2}$ s. The flow corresponds to a Womersley number of 3.0. We link the expansion of the vessel to the pressure via $\Delta E = \frac{(1-\sigma ^2)R^2}{Y h}(p - P_0)$, where $P_0$ is the pressure at which $\Delta E = 0$. We use three different levels of grid refinement to study this case. Here we chose the lattice spacing, $\Delta x$, such that: $R = 50 \Delta x$, $100 \Delta x$ and $200 \Delta x$. The time step was chosen for each level of grid refinement to ensure that the Young’s modulus of the vessel was consistent between cases. We also examined two levels of applied pressure gradient amplitude: $k_p = -50$ Pa/m and $-150$ Pa/m. We compare the numerical results to those obtained from the analytical solutions. In particular we will examine the axial velocity obtained along the centreline of the cylindrical test domain and across the radius at a plane in the centre of the domain. As the radius of the cylinder is 3 mm, and the extension was observed to be relatively small, we decided to use a boundary velocity ratio of $F = 0.85$. All simulations were conducted on the SuperMUC-NG supercomputer situated at the Leibniz Supercomputing Centre, Germany (https://doku.lrz.de/display/PUBLIC/SuperMUC-NG). We provide details on the computational configurations used for our simulations in the Appendix.

**Figure 3:**
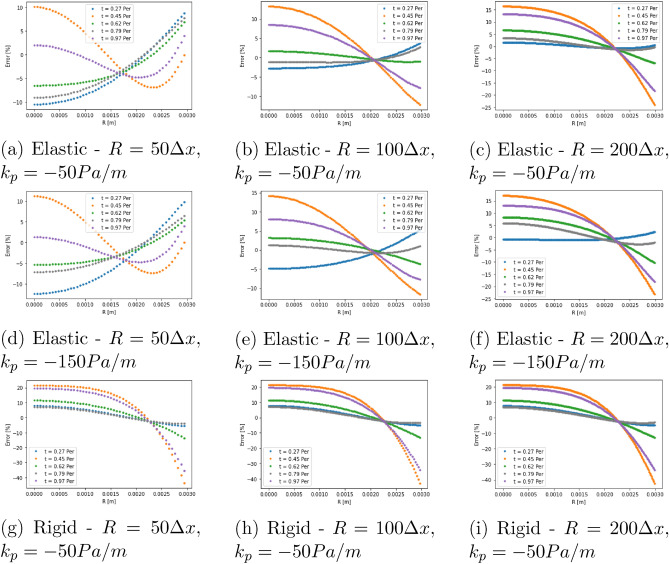
Relative error at the centre plane of the test cylinder comparing the current elastic wall model to the elastic wall analytical equations for the cases of (**a**)–(**c**) $k_p = -50$ Pa/m and (**d**)–(**f**) $k_p = -150$ Pa/m. (**g**)–(**i**) compare the rigid wall model to the elastic wall analytical equations for $k_p = -50$ Pa/m. Note the significantly larger error at the walls compared to the equivalent results for the elastic wall model, In all cases, the centre of the cylinder is at $R=0.0$ m and the edge at $R=0.003$ m.

**Figure 4:**
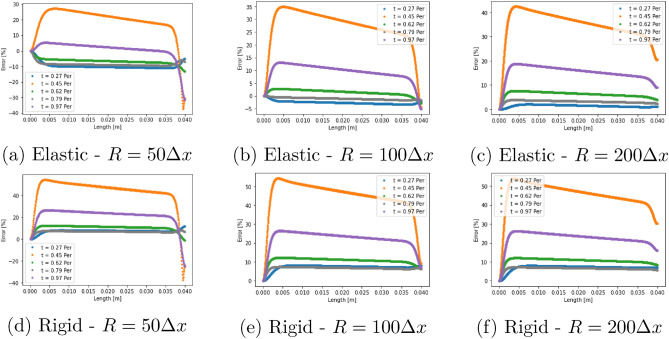
Relative error along the central axis of the test cylinder comparing (**a**)–(**c**) the current elastic wall method and (**d**)–(**f**) the rigid wall method to the elastic wall analytical solution for the case of $k_p = -50$ Pa/m. The flow inlet was at $L=0.0$ m and the outlet at $L=0.04$ m.

In Fig. 3 we compare the relative error of the central plane velocity profiles for the two pressure gradient cases at each of the geometric resolutions examined. Throughout this work, we have defined the error with the following function: $error[\%] = \frac{100(abs(V_{LBM}) - abs(V_{Theory}))}{max(abs(V_{Theory}))}$, where $V_{LBM}$ and $V_{Theory}$ represent the calculated and analytical longitudinal velocity along the plane of interest. In our results, we allowed the simulation to overcome initialisation effects and then compared the calculated profiles at 5 stages within an oscillation period. Generally speaking, our model is able to replicate the expected analytical results with less than 10% error, with the greatest error being observed at the time steps with the lowest flow velocity magnitudes in the central plane where relative errors can be magnified. It should also be noted that there is varying error behaviour as the resolution of the cylinder is increased which may be related to the choice of boundary velocity ratio providing a better approximation of the elastic wall flows in some circumstances. In Fig. 4, we demonstrate similar trends for the axial velocity recorded along the central axis of the cylinder for the case of $k_p = -50$ Pa/m. In several of the error plots presented in Figs. 3 and 4, the largest errors occur at $t=0.45~period$ and $t=0.97~period$. These two cases represent the instantaneous flow profiles with the lowest velocity magnitudes and are where any absolute errors—perhaps induced by the choice of boundary velocity ratio—present within our model are magnified by the relative perspective presented by the plots.

For the case $k_p=-50$ Pa/m we also generated results when a rigid wall assumption is in place. Here we use the well-known LBM bounceback condition to represent the solid walls. Error profiles at the central plane are presented in Fig. 3 whilst comparison to the central axis velocity is provided in Fig. 4. In both of these cases, the error observed when rigid walls are enforced is notably greater than that seen with the our proposed elastic wall condition. These collective results indicate that we are able to capture the key flow results associated with an elastic wall better using our model than can be achieved with a rigid wall implementation.

**Figure 5:**
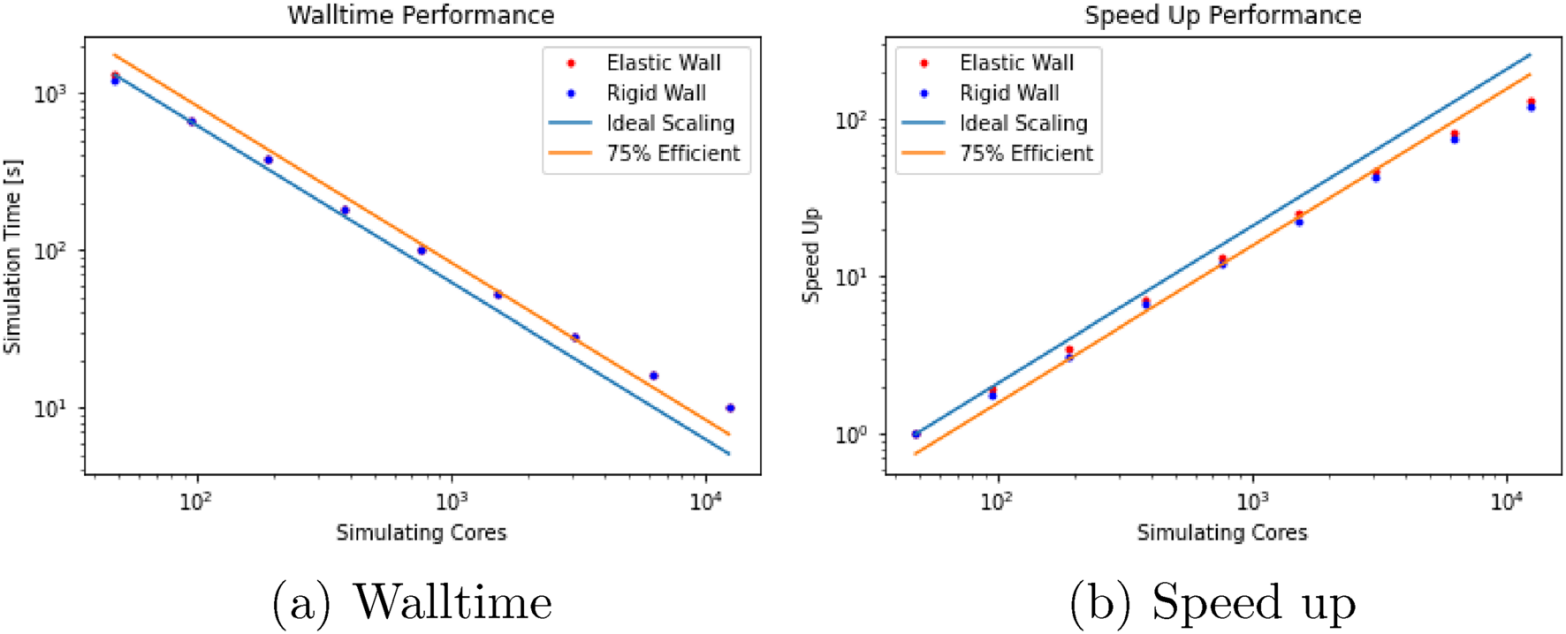
Strong scaling results of HemeLB using our elastic wall implementation and the bounceback rigid wall model. The walltime and speed up results are virtually identical for the two cases. We provide lines of ideal performance and 75% efficiency as a guide.

To evaluate the performance change brought about by the use of our model for elastic walls, we conducted a small strong scaling study using the $R = 100\Delta x$ domain. In this comparison, we measured the simulation time to complete 5000 steps with both our elastic wall implementation and rigid walls using the bounceback method. This was conducted using between 1 and 256 nodes on SuperMUC-NG (48–12,288 cores). As can be seen in Fig. 5, the observed walltime and relative speed up to complete the simulation is almost identical for the two boundary implementations. This demonstrates that our model is able to better capture the expected flow in elastic vessels without a loss in performance compared to a rigid wall model. This shows that our approach overcomes the argument often used to justify the use of rigid wall models in a vascular simulation.

## Model application

**Figure 6:**
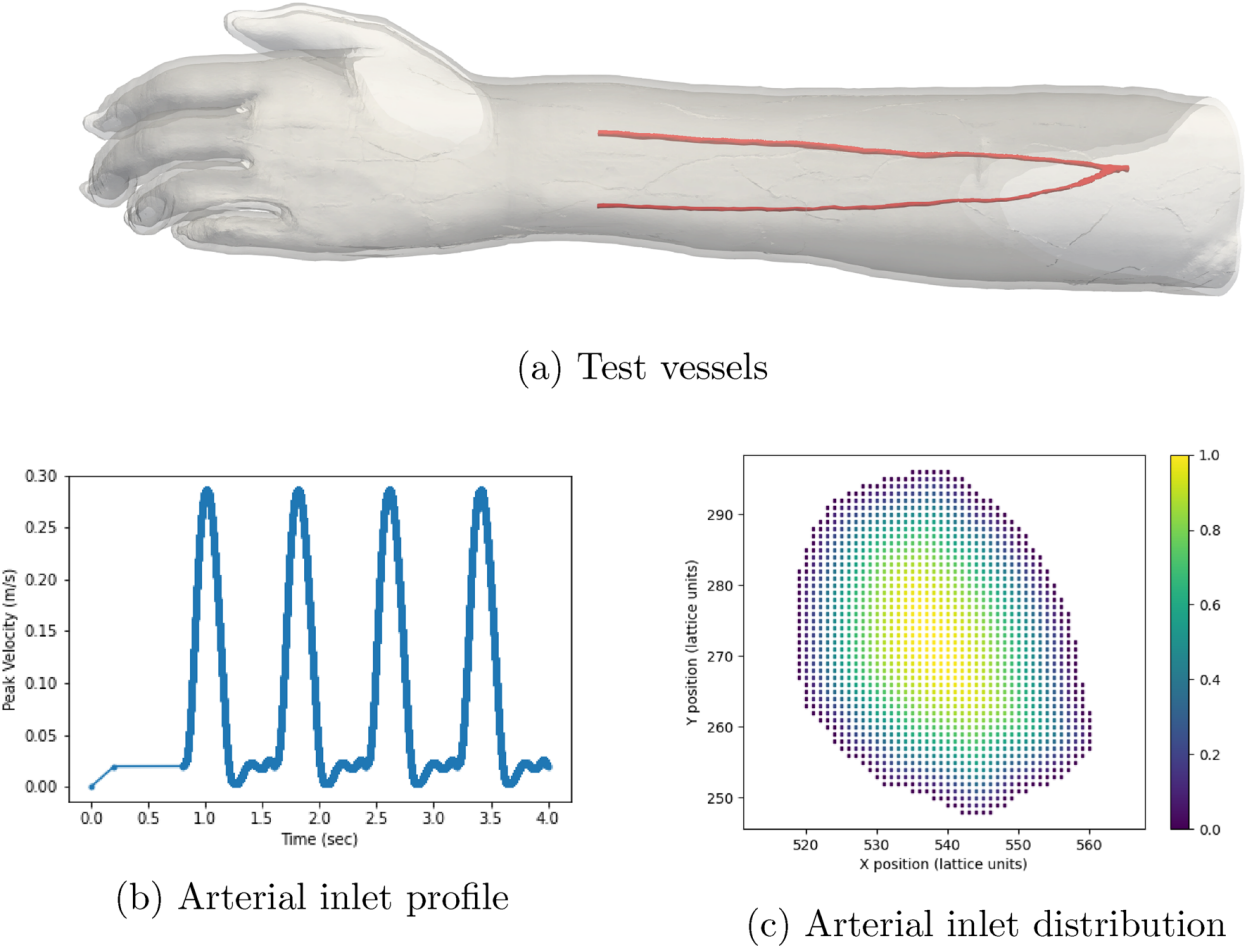
Simulation domain and inlet conditions used in for the demonstration arterial studies. Here we simulated four heartbeats of pulsatile flow within the vessels using our elastic wall model and a rigid wall model. We also compared the behaviour at two different size scales to examine the effect of different boundary velocity ratios. (**a**) Illustrates the original flow domain itself within the left forearm. (**b**) Details the flow velocity provided to the inlet of the arterial geometry for the forearm flow cases. The first 0.8 s of the flow represents an initial warm-up period of flow within the system. (**c**) indicates the distribution of scaling weights applied to the flow velocity at the inlet of the arterial geometry.

**Figure 7:**
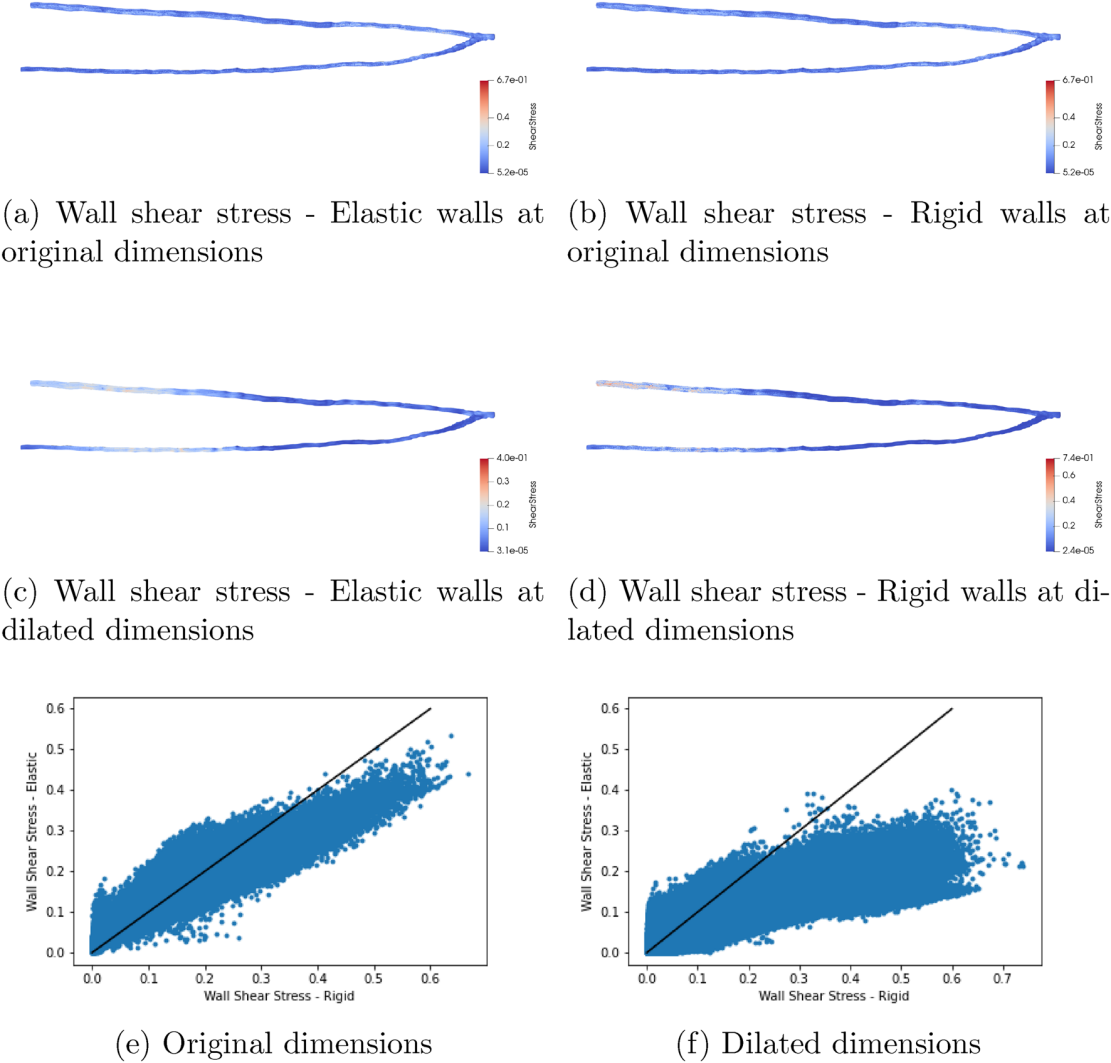
Wall shear stress fields for the arterial domain in their original (**a**, **b**) and dilated (**c**, **d**) dimensions after approximately 60% of the total simulation time. In figures (**e**, **f**) we present a pointwise comparison of instantaneous wall shear stress seen across the full domain of the arterial geometry in the rigid and elastic walled cases, the solid line represents the identity line. The shape of the plot—generally recording higher wall shear stress in the rigid walled case, especially at higher stress magnitudes—is consistent with that generated in other work using a fully coupled elastic wall model^28^. This result further demonstrates that our model is able to generate the elastic wall behaviour expected from a fully coupled approach whilst retaining the performance characteristics of a rigid wall implementation. The greater discrepancy between the rigid and elastic shear stresses in the dilated case also indicates how the choice of a larger boundary velocity ratio provides a more elastic vessel.

In this section we demonstrate the behaviour of our model in patient specific vessels. The domain we are studying consists of the radial and ulnar arteries of the left forearm (see Fig. 6). To drive the flow, we provide a velocity profile to the inlet plane that is representative of the pulsatile flow within the vessels at this point in the vasculature, our profile contains an initial warm-up period before completing four heartbeat cycles (Fig. 6b). Since our model is in 3D, we provide a scaling factor at each point in the inlet plane to generate a Poiseuille-like flow profile within the vessel (Fig. 6c). Fixed pressure conditions^11^ were applied to the outlets. Based on the geometry of the vessels we have chosen a value for the boundary velocity ratio of $F = 0.025$. To replicate larger vessels, we have taken the same domain and adjusted the size of the lattice spacing to dilate the vessels by a factor of approximately four. Whilst not representative of a particular vessel it carries the characteristics of a patient-specific geometry and allows us to present results representative of a more flexible vascular domain. In this second case we have used a value of $F = 0.5$. Simulations of both domains with elastic and rigid walls were conducted on SuperMUC-NG; the computational details for these simulations are presented in the Appendix. Figure 7 illustrates the wall shear stress fields observed approximately 60% through the simulation time for both size scales. In both cases the lower shear stress observed in the elastic wall cases is consistent with observations made in other numerical studies of patient specific vessels^28,29^. This is made more explicitly clear when we compare the local, instantaneous wall shear stress between the rigid and elastic cases in Fig. 7e, f. Here the shape of our agreement plot of instantaneous wall shear stress is very similar to that presented in McGah et al.^28^ for time-averaged wall shear stress. This again demonstrates that our model is able to effectively capture behaviour expected from a fully coupled elastic wall model.

## Discussion

When compared to other sources of uncertainty related to measuring and validating blood flow in personalised geometries—particularly those associated with clinical measurement techniques such as ultrasound or MRI, the error indicated by our simple model is of a similar order of magnitude. For example, Merkx et al.^30,31^ record a significant difference between the diameters recorded using an MRI technique and ultrasound and state that the MRI technique may overestimate vessel diameters by 38% potentially in part due to the sensitivity of the technique to patient position. Furthermore, Merkx et al.^31^ note that the diameter of the radial artery may vary by up to 4–7% during day-to-day function. Image analysis techniques used to assess medical images can generate similar levels of variation and uncertainty^32^. The uncertainty in velocity measurements from MRI is further discussed in Bruschewski et al.^33^. Indeed, Brindise et al.^34^ summarises the challenge in validating against MRI derived data as “A major challenge for any multi-modality study that uses in vivo measurements is that no ‘ground-truth’ flow field can be established”. Keeping these factors in mind, the errors presented from our model compared to those generated with a rigid wall approximation are more than acceptable.

One advantage of including elastic walls to a model is the inclusion of a windkessel effect that can smooth flow changes. We implemented a sinusoidal driving velocity to the $R = 50 \Delta x$ cylinder and observed that, whilst initialisation transients lasted slightly longer in our model than with the rigid wall implementation, they possessed a smaller amplitude. Once flow was established, both models illustrated the same behaviour. This indicates that our boundary condition can provide a limited windkessel effect to flow when compared to a rigid wall boundary condition.

Further development of this model would be best focussed on how its implementation could be improved to effectively study domains with a greater spread in vascular diameters and resolution whilst retaining the locality of the implementation. As noted above this could be achieved with a local specification of the boundary velocity ratio. How a global (or regional) value for this term could be better tuned to different flow scenarios would also be of interest to the study of large-scale vascular structures. From the definition of the boundary velocity ratio, we would anticipate that vascular regions requiring a more flexible wall—such as larger arterial vessels or aneurysms—would demand a value of *F* closer to unity. Stiffer regions would require smaller values of the boundary velocity ratio, with $F=0$ being the limit for a rigid wall.

## Conclusion

In this paper we have presented a boundary condition that allows key features of elastic walled flow such as velocity profiles near walls and wall shear stress variations to be captured without the need to implement a complex computational coupling with a solid mechanics solver. This has been implemented within the LBM-based, 3D blood flow simulator HemeLB. Our boundary condition is based on the application of a slip velocity at the wall of the domain that represents the flow at that physical location if an elastic wall was extended beyond it. This is calculated through a boundary velocity ratio that can be estimated based on the physical properties of the simulated vessel and the expressions for Womersley flow in an elastic cylinder. Whilst our model does not, and is not intended to, perfectly capture the analytical flow profiles expected within an elastic walled cylinder, it is significantly more accurate than results found using a rigid wall assumption. This was achieved with no loss of computational performance compared to the implementation of the bounceback boundary condition for rigid walls in HemeLB. These results indicate that our model would represent an effective return on the investment of implementing it within other LBM-based vascular simulation tools. Although we have discussed the boundary velocity ratio as a global parameter within this paper, there is no fundamental reason why it could not be tuned locally within a geometry of widely varying vessel diameters such as a whole human vascular tree.

With a view towards the development of a virtual human, our model will now permit HemeLB to conduct efficient, high resolution, 3D blood flow simulations with the effect of elastic walls included. Not having to support an explicit coupling for the solid mechanics of the vessel walls will reduce the communication burden of the simulation and allow resources to be deployed to other components of a virtual human model.

## Supplementary Information

Supplementary Information.
