# Supplementary material for: An efficient, localised approach for the simulation of elastic blood vessels using the lattice Boltzmann method
Source: Sci Rep. 2021 Dec 20;11:24260. doi: 10.1038/s41598-021-03584-2 (PMC8688478; doi:10.1038/s41598-021-03584-2)
Supplement: Supplementary file 1 — Supplementary Information. [file 41598_2021_3584_MOESM1_ESM.pdf]

An efficient, localised approach for the simulation of elastic blood vessels using the lattice Boltzmann method

McCullough, J.W.S.[1] and Coveney, P.V.*[1,2]

[1] *Centre for Computational Science, Department of Chemistry, University College London, UK*
[2] *Informatics Institute, University of Amsterdam, Netherlands*

September 21, 2021

Appendix

Table A1: Simulation Parameters

**Test**	Cylinder (R=50Δx)	Cylinder (R=100Δx)	Cylinder (R=200Δx)	Arteries - Original	Arteries - Dilated
Δx [**m**]	6.0e-5	3.0e-5	1.5e-5	5.0e-5	2.1e-04
Δt [**s**]	1.10e-05	5.48e-06	2.74e-06	5.0e-6	1.0e-4
τ	0.537	0.573	0.646	0.527	0.527
**Steps**	1,000,000	2,000,000	4,000,000	660,000	33,000
**Lattice sites**	5,195,466	41,596,265	332,841,364	6,128,855	6,128,855
**Cores**	3072	6000	12000	2400	2400

All simulations were run on the SuperMUC-NG supercomputer (`https://doku.lrz.de/display/PUBLIC/SuperMUC-NG`) situated at the Leibniz Supercomputing Centre, Germany. This machine uses Intel Skylake processore (Xeon Platinum 8174) with 48 CPU cores per node. Simulations were run using the full complement of cores on each node. Nodes are connected with an OmniPath interconnect configured in an island layout. HemeLB was compiled using the default 2019 versions of Intel C++ compilers and MPI.

The version of HemeLB used for this study can be obtained from `https://github.com/UCL-CCS/HemePure`.
